# FUNDC2, a mitochondrial outer membrane protein, mediates triple-negative breast cancer progression via the AKT/GSK3β/GLI1 pathway: FUNDC2 mediates TNBC progression via the AKT/GSK3β/GLI1 pathway

**DOI:** 10.3724/abbs.2023142

**Published:** 2023-09-11

**Authors:** Liyang Yin, Renxian Cao, Zhuoqing Liu, Gang Luo, Yu Li, Xiaolong Zhou, Xiguang Chen, Ying Wu, Jun He, Xuyu Zu, Yingying Shen

**Affiliations:** 1 The First Affiliated Hospital Cancer Research Institute Hengyang Medical School University of South China Hengyang 421001 China; 2The Nanhua Affiliated Hospital Department of Spine Surgery Hengyang Medical School University of South China Hengyang 421001 China

**Keywords:** FUNDC2, GLI1, triple-negative breast cancer (TNBC), therapeutic target

## Abstract

Triple-negative breast cancer (TNBC) lacks effective therapeutic targets and has a poor prognosis, easy recurrence and metastasis. It is urgent and important to explore TNBC treatment targets. Through mass spectrometry combined with qRT-PCR validation in luminal A cells and TNBC cells, high-content screening and clinical sample analysis, FUNDC2 was discovered as a novel target. The function of the outer mitochondrial membrane protein FUNDC2 in breast cancer is still unclear. In this study, we find that FUNDC2 expression in TNBC tissues is significantly higher than that in luminal subtype breast cancer tissues. *FUNDC2* silencing in TNBC cells significantly reduces cell proliferation, migration and invasion. As demonstrated *in vivo* using subcutaneous tumor xenografts in mice, FUNDC2 suppression significantly inhibits tumor growth. The underlying mechanism might be mediated by inactivating its downstream signal AKT/GSK3β and GLI1, a key factor of the Hedgehog signaling pathway. Therefore, FUNDC2 may promote TNBC progression and provide a therapeutic target for treating TNBC.

## Introduction

Breast cancer has overtaken lung cancer as the tumor with the highest incidence rate around the world [1]. Currently, cancer research is one of the hottest spots. Triple-negative breast cancer (TNBC), the leading cause of breast cancer-related death in females worldwide, is an aggressive breast cancer subtype with a poor prognosis compared with ER-positive and HER2-positive subtypes [ 2‒ 4] . It accounts for 10%‒20% of newly diagnosed breast tumors, which is more likely to relapse than other subtypes and lacks effective therapeutic targets [ 5‒ 7] . It is still difficult to establish a molecular target for TNBC, and the prognosis for TNBC patients is generally unfavorable [ 8, 9] . The molecular mechanisms underlying TNBC remain a major hurdle in treating this disease, despite great efforts have been put into this research. Therefore, effective therapeutic strategies and new targets are urgently needed for TNBC.

FUNDC2 (FUN14 domain-Contains 2, also known as HCBP6) is the homologous protein of FUNDC1 [10], which regulates platelet function via the AKT/GSK3β/cGMP signaling pathway [ 11, 12] . Recently, it was reported that FUNDC2 promotes liver tumorigenesis by inhibiting MFN1-mediated mitochondrial fusion [13]. However, studies on FUNDC2 in breast cancer have not been reported thus far, and the potential mechanism by which FUNDC2 promotes the development of TNBC is still unclear.

The Hedgehog-GLI (HH-GLI) pathway is involved in the progression of multiple types of cancers, including breast cancer, gastric cancer and osteosarcoma [ 14‒ 17] . Dysregulation of the HH-GLI pathway can affect the development of breast cancer [18]. The Hedgehog signaling pathway mediates the progression of noninvasive breast cancer to invasive breast cancer [19]. GLI1 is the final effector molecule of the HH-GLI signaling pathway. The basal expression levels of GLI1 and GLI2 downstream of the Hh ligand in TNBC are higher than those in hormone receptor-positive (HR+) breast cancer, and the prognosis of breast cancer patients with high GLI1 expression is poor [20]. GLI1 activation is one of the mechanisms by which estrogen exposure promotes the proliferation and epithelial-mesenchymal transition of breast cancer stem cells [21], and GSK3β inactivation can inhibit the activation of GLI1 [22]. Several previous studies have shown that AKT activation promotes GLI1 expression in other tumors [ 23, 24] . Nevertheless, it remains unclear whether the HH-GLI1 pathway plays a vital role in the effect of FUNDC2 on TNBC.

In the present study, we discovered a novel target, FUNDC2, by comparing luminal A subtype breast cancer and TNBC. The effectiveness of FUNDC2 in TNBC was evaluated *in vitro* and *in vivo*, and the underlying mechanism was elucidated. We found that the Hedgehog pathway, which is closely related to breast cancer, was significantly inhibited after silencing of *FUNDC2* by whole genome microarray.

## Materials and Methods

### Cell lines and antibodies

The luminal-A subtype human breast cancer cell line MCF7 and TNBC cell lines MDA-MB-231 and HS-578T were obtained from the National Collection of Authenticated Cell Cultures (Shanghai, China). All cell lines were cultured in DMEM (Gibco, Grand Island, USA) supplemented with 10% fetal bovine serum (FBS) and 1% penicillin streptomycin solution at 37°C with 5% CO _2_.

Antibodies against phospho-AKT (S473; #4060) and phospho-GSK3β (S9; #9323) were purchased from Cell Signaling Technology (Beverley, USA). Antibodies against GSK 3β (22104-1-AP), AKT (10176-2-AP), GLI1 (66905-1-Ig), and β-actin (66009-1-Ig) were obtained from Proteintech (Wuhan, China). Anti-FUNDC2 antibody (H00065991-B02P; Abnova, Taibei, China) was used for immunoblotting, and anti-FUNDC2 antibody (HPA059129; Atlas Antibodies AB, Bromma, Sweden) was used for immunohistochemistry analysis.

### Isobaric tags for relative and absolute quantification (iTRAQ)

iTRAQ is a technique for relative and absolute quantification based on isoheavy isotope labeling *in vitro*. According to a previous report [25], iTRAQ labeling was performed. Eight isotope reagents (113, 114, 115, 116, 117, 118, 119 and 121) were used to label the amino group or amino group of the lysine side chain of the polypeptide. MDA-MB-231 cells were divided into three groups, A1, A2 and A3, which were labeled with 113, 114, and 115, respectively. MCF7 cells were divided into three groups, B1, B2 and B3, which were labeled with 118, 119 and 121, respectively. The protein expression levels of the six samples were compared simultaneously by high-resolution mass spectrometer series analysis.

### Data comparison and analysis

The Proteome Discoverer 2.1 software was used to submit the raw spectra generated by Q Exactive Plus mass spectrometer to the MASCOT 2.5 server for database searching using the built-in tools. The selected database for this study was Uniprot-HomoSapiens. Subsequently, the search results obtained from the MASCOT server were transferred back to the Proteome Discoverer 2.1 software. The data was filtered based on the FDR<0.01 criterion to obtain highly reliable qualitative results.

### Gene Ontology analysis

Blast2GO was utilized for the Gene Ontology (GO) annotation of differentially expressed proteins, involving several steps: sequence comparison, term extraction, GO annotation, and supplementary annotation explanations. Firstly, the proteins to be aligned were compared against the NCBI BLAST+(ncbi-blast-2.2.28+-win32.exe), and the top 10 sequences with an E-value ≤1×10 ^-3^ were further analyzed. Subsequently, the Blast2GO Command Line was employed to perform the annotation of target proteins. During the annotation process, the Blast2GO Command Line evaluated the authenticity and reliability of GO terms comprehensively, and assigned the extracted GO terms from the mapping process to the target protein sequences. After completing the annotation, InterProScan was used to search the EBI database for conserved domains matching the target proteins. The functional information related to motifs was then annotated to the target protein sequences. Additionally, the ANNEX module was executed to complement the annotation information and establish connections between different GO categories, aiming to improve the accuracy of annotation.

### KEGG

KEGG ( https://www.genome.jp/kegg/ or http://www.kegg.jp/) serves as an encyclopedia of genes and genomes, aiming to assign functional meanings to genes and genomes at both the molecular and higher levels. The primary objective of the KEGG database project is to provide comprehensive functional annotations for genes and genomes. Molecular level functions are stored in the KO (KEGG Orthology) database, where each KO is defined as a functional ortholog of genes and proteins. Higher level functions are represented by networks of molecular interactions, reactions, and relationships, which are showcased in the form of KEGG pathway maps, BRITE hierarchies, and KEGG modules.

### High-content screening

MDA-MB-231 cells were infected with the mixed packaging vector of lentivirus, and shRNAs for 20 genes ( Supplementary Table S1).

For proliferation assay, cells were seeded in 96-well plates at 1500 cells per well and then cultured at 37°C with 5% CO _2_ for 5 days. The cells were photographed and counted according to the fluorescent protein signal expressed by the cells after infection for 1, 2, 3, 4 and 5 days. A Celigo Image Cytometer (Nexcelom, Essex, UK) was used to scan the plates at the same time each day. The cells were set as the experimental group, Ctrl (negative control) group and PC (positive control) group. The lentivirus used was purchased from GeneChem (Shanghai, China). Curves were drawn according to the changes in the number of cells in each group, and proliferation was observed after gene silencing by adjusting the input parameters of Analysis Settings. On day 5 of cell proliferation, the fold change in the cell count in the Ctrl group was compared with that in the experimental group.

For wound-healing assay, cells were digested from each experimental group in logarithmic growth phase with trypsin, resuspend in complete medium, and counted. Seeding density was determined based on cell size (most cells are plated at 50,000 cells/well) to achieve over 90% confluence the next day. Cells were cultured at 37°C in a 5% CO _2_ incubator (3 wells per group, 100 μL/well). The next day, low serum medium was replaced. A scratch maker was used to create a vertical scratch at the center of the 96-well plate bottom. Gently wash 2‒3 times with serum-free medium, add low serum medium ( *e*. *g*., 0.5% FBS), and take images at 0 h. Cells were cultured at 37°C in a 5% CO _2_ incubator. Based on healing progress, appropriate time was chosen for Celigo imaging and migration area was analyzed using Celigo. After 24 h of cell migration, the migration area inhibition rate in the Ctrl group was compared with that in the experimental group. To identify genes with differential expression, log2(fold change) values greater than 1.5 were used.

### RNA interference and overexpression plasmids

Cells were transfected with siRNA (100 nM) or shRNA synthesized by GenePharma (Suzhou, China). siRNAs were transfected into cells using Lipofectamine RNAiMAX Transfection Reagent (Thermo Fisher Scientific, Waltham, USA) for 48 h. The sequences of siRNAs and shRNAs are shown as follows: negative control (NC) siRNA: 5′-UUCUCCGAACGUGUCACGUTT-3′; FUNDC2 siRNA-1: 5′-CAGGUUUCAUAUUCCAGAATT-3′; FUNDC2 siRNA-2: 5′-UCCGUAAGAGCAAUCAGAUTT-3′; FUNDC2 siRNA-3: 5′-AUCUGGACCUUCAGCAGAATT-3′; NC shRNA: 5′-TTCTCCGAACGTGTCACGT-3′; and FUNDC2 shRNA: 5′-TCCGTAAGAGCAATCAGAT-3′. shRNA was packaged with lentivirus to transfect cells for 72 h. Positive cells were screened after two weeks of treatment with puromycin.

Full-length coding sequences were obtained from the NCBI Gene Bank for overexpression. To clone human AKT and GLI1 coding sequences into the pcDNA3.1 expression vector, full-length PCR amplifications were performed. Furthermore, the vector DNA plasmids and the overexpression plasmids were transfected into cells at a rate of 2 μg/well for 48 h. Transfection of cells was performed using Lipofectamine 3000 (Thermo Fisher Scientific) following the manufacturer’s instructions.

### RNA extraction and quantitative PCR

qRT-PCR was conducted according to our previous study [26]. Total RNA was extracted from tissues and cells with Trizol reagent (Cwbio, Taizhou, China). Reverse transcription of RNA into cDNA was performed with the RevertAid First Strand cDNA Synthesis kit (Thermo Fisher Scientific). qRT-PCR was performed using a one-step RNA PCR kit (TaKaRa, Dalian, China). The primers used for quantitative PCR are listed in Supplementary Table S2 and were synthesized by Sangon (Shanghai, China). The PCR profile was as follows: 1 cycle, 95°C for 5 min; 40 cycles, 95°C for 15 s, 55°C for 30 s, and 70°C for 30 s. *GAPDH* was used as a negative control, and the 2 ^–ΔΔCT^ method was used to calculate the relative expression.

### Western blot analysis

Cells or tissues were collected for total protein extraction. Cut tissue samples into pieces, grind into a fine powder using liquid nitrogen until no visible solids remain, collect the supernatant, and centrifuge. RIPA buffer (Beyotime, Shanghai, China) was used for protein lysis at 4°C for 30 min. Then, 10% SDS-PAGE was performed and the proteins were transferred onto PVDF membranes (Merck Millipore, Molsheim, Germany). Membranes were blocked with 5% skim milk for 90 min, and then incubated with the primary antibodies overnight at 4°C. After being washed with TBST three times (5 min/wash), the membranes were incubated with HRP-conjugated secondary antibodies (Proteintech, Wuhan, China) at room temperature for 1 h. The PVDF membranes were washed with TBST 3 times (10 min/wash) and the blots were detected with an ECL Detection System (Merck Millipore). The ratio of band intensity of the target protein compared to β-actin or GAPDH was used to represent relative protein expression levels. Densitometric analysis was performed with ImageJ (NIH, Bethesda, USA).

### Hematoxylin and eosin (HE) staining

Immerse sections sequentially in two baths of xylene, two baths of absolute ethanol, and one bath of 75% ethanol for 5 min each, and rinse gently with clean water. Incubate sections in sediment-free hematoxylin solution for approximately 4 min, followed by differentiation in hydrochloric acid solution. Then apply ammonia water to blue, wash in water. Following graded alcohol dehydration, stain sections in eosin solution for 5 min. After sequential immersion in 3 baths of absolute ethanol and 2 baths of xylene for 5 min each, mount with neutral resin. Images were taken under the microscope.

### Immunohistochemistry (IHC) and clinical data

The immunohistochemical experiment was performed using ElivisionTMsuper HRP (Mouse/Rabbit) IHC kit ( MXB Biotechnologies, Fuzhou, China) according to the kit’s instructions, and immunohistochemical results for all indicators were scored by two pathologists [27]. The staining intensity and tumor cell proportion scores were as follows: 0 (no tumor cell positivity), 1 (no staining, <10% positive tumor cells), 2 (light brown, 10%‒50% positive tumor cells), 3 (yellow brown, 50%‒75% positive tumor cells), and 4 (brown, >75% positive tumor cells). Staining index (SI)= staining intensity score × proportion of positive tumor cells, judged by their scores: <4 points, (‒); 4‒8 points, (+); 8‒12 points, (++); and 12‒16 points, (+++). Qualitative and semiquantitative results were obtained for coloring intensity. According to the above description, at least 10 high-powered fields were evaluated for IHC signals.

### Colony formation assay

At the logarithmic growth stage, cells were digested with 0.25% trypsin when the cells were in good condition. After centrifugation, the cells were suspended again and then blown into a single-cell suspension. A total of 1000 cells/well were seeded into 6-well plates. Cells were cultured for 15 days at 37°C with 5% CO _2_, and cell culture was terminated when visible clones appeared. The supernatant was discarded, and cells were stained with 0.5% crystal violet. Then photos were taken after staining. Finally, the number of colonies was counted and the colony formation rate was calculated.

### Cell viability assay

Cells were treated as described above. A total of 1500 cells were seeded into each well of 96-well plates. The cells were cultured in the cell incubator for 0, 24, 48, 72 and 96 h at 37°C with 5% CO _2_. Then, 20 μL MTS (Promega, Madison, USA) was added to each well and incubated for 3 h. The cell growth curve was drawn based on the optical densities measured at 490 nm with a microplate reader (iMark; Bio-Rad, Hercules, USA).

### Cell migration and invasion assay

For cell migration assay, 300 μL of cell suspension (1×10 ^5^ cells) without FBS was added to each upper chamber of transwell inserts (Corning, Corning, USA), and 500 μL of medium containing 10% FBS was added to the lower chamber. For cell invasion assay, matrigel (Corning) was used to coat the upper chamber of the transwell inserts. Five hundred microliters of medium containing 10% FBS was added to the lower chamber, and 300 μL of cell suspension (2×10 ^5^ cells) without FBS was added to the upper chamber. The plates were cultured at 37°C with 5% CO _2_ for 48 h. Migrated or invaded cells were washed with PBS, fixed for 5 min in methanol, and stained with 0.05% crystal violet solution. Then, photos were taken after staining. Finally, the number of migrated or invaded cells was counted.

### Tissue microarray (TMA)

Two tissue microarrays were included in this study. The first tissue microarray contains two parts. A portion was collected from 94 breast cancer (80 luminal subtypes and 14 TNBC) and 33 tissues adjacent to carcinoma in the Department of Pathology, the Second Affiliated Hospital of University of South China. The other part was a TMA (Cat No. BR487c) containing 44 TNBC and 2 luminal subtypes purchased from Xi’an Ailina Biotechnology Co., Ltd (Xi’an, China). The second tissue microarray was collected from 78 TNBC and 78 tissues adjacent to carcinoma in the Department of Pathology, the First Affiliated Hospital of University of South China. The tissue sections were scanned and imaged by a panoramic section scanner (TissueFAXS SL Spectra S, TissueGnostics, Austria), and the tissue microarray information was analyzed by Quant Center 2.1 analysis software. The H-Score was calculated by the Densito Quant module.

### Tumor xenograft models

The Animal Ethics Committee of the University of South China approved all animal experiments (USC202209XS04). BALB/c female nude mice (18–20 g, 5 weeks old) were obtained from Hunan SJA Laboratory Animal Co., Ltd (Changsha, China) and randomly assigned into two groups: (1) the NC group, injected with MDA-MB-231 cells transfected with control lentivirus; and (2) the shFUNDC2 group, injected with MDA-MB-231 cells transfected with FUNDC2-shRNA lentivirus; 1×10 ^6^ cells mixed with matrigel were injected into a fat pad of 4- to 6-week-old BALB/C nude mice. Body weight and tumor volume were monitored 7 days after injection, and tumor volume was calculated as follows: tumor volume=(long diameter×short diameter ^2^×0.52). At the end of experiment, tumor tissues were collected for subsequent analyses.

### Statistical analysis

Statistical analysis of cell phenotype and qRT-PCR was performed with GraphPad Prism 6.0 software, and differences between groups were compared with Student’s *t* test. qRT-PCR data were quantified and analyzed using the 2 ^–ΔΔCT^ method. *P*<0.05 was considered statistically significant. *P*<0.01 was considered to be highly significant.

SPSS 24.0 software was used for clinical statistical analysis, and the Mann-Whitney U (M-U) test was used for ranked data comparison. Multivariate Kruskal-Wallis tests were used to compare the difference among groups. The Spearman correlation coefficient was used for correlation analysis. Fisher’s exact test was used to determine if there are nonrandom associations between two categorical variables. *P*<0.05 was considered statistically significant.

## Results

### Differential protein expression between the TNBC subtype (MDA-MB-231 cell line) and luminal A subtype (MCF-7 cell line) of breast cancer

**Figure 1:**
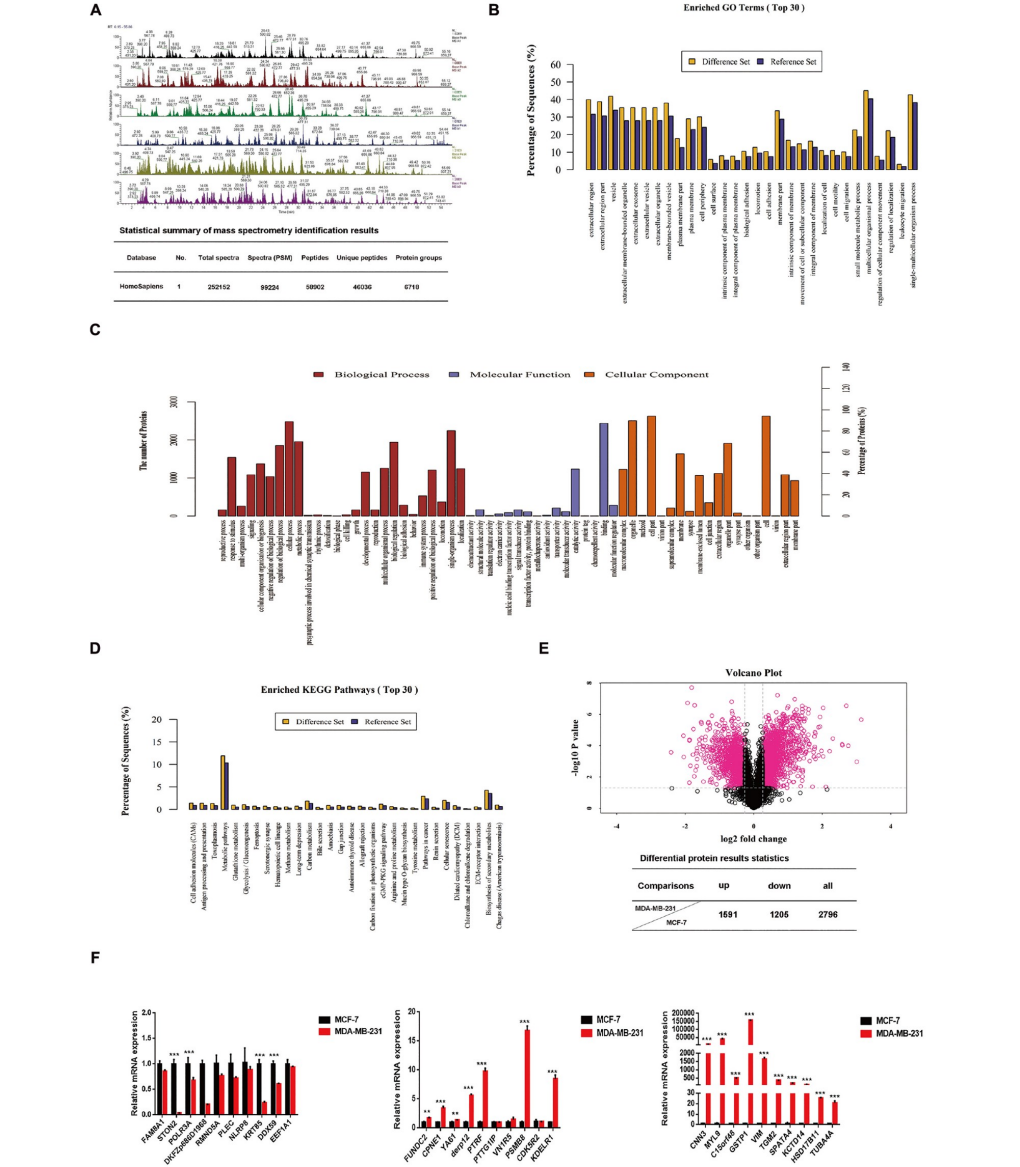
Figure 1 Differential protein expression between MDA-MB-231 and MCF-7 breast cancer cell lines (A) Top: analysis of differential protein expression between MDA-MB-231 (TNBC cells, A1–A3) and MCF7 (luminal A cells, B1–B3) cells by high-performance liquid chromatography (HPLC). Bottom: statistical summary of mass spectrometry identification results. Spectra (PSM, Peptide Spectrum Match). (B) The horizontal axis represents the significance level of a particular GO term protein enrichment, and the vertical axis represents the percentage of sequences. (C) GO annotation analysis of differential protein classification. The vertical axis represents the number of differentially expressed proteins, and the horizontal axis represents the differentially expressed proteins at the level of biological process, molecular function, and cell composition. (D) KEGG analysis of differentially expressed proteins. The horizontal axis represents the metabolic and signal transduction pathways involved in differential proteins, and the vertical axis represents the proportion of peptides. (E) Volcano plot showing the significant difference between the two groups of samples. The abscissa is the fold change, and the ordinate is the P value (top). The table shows the differential protein results (bottom). fold change>1.2, P<0.05. (F) The expression of 30 differentially expressed genes between MCF-7 and MDA-MB-231 cells was detected by qRT-PCR (due to the large difference in fold changes, it is divided into three figures). *** P<0.001.

iTRAQ proteomics was used to explore the differential proteins in luminal A cells and TNBC cells. A total of 46,036 unique peptides were screened ( Figure 1A). By qualitative analysis of differential proteins using Proteome Discoverer 2.1 software, combined with GO annotation and enrichment analysis ( Figure 1B,C) and KEGG analysis ( Figure 1D), Fisher’s exact test was used to evaluate the significance level of protein enrichment of the top 30 GO terms. The differentially expressed proteins were mainly involved in the processes of multicellular organisms, cell adhesion and cell movement and were mainly involved in metabolic pathways and tumor-associated pathways. The differential protein expression between the two groups of unique proteins was analyzed, and a total of 2796 differentially expressed proteins were found, including 1591 upregulated proteins and 1205 downregulated proteins in MDA-MB-231 cells. MCF-7 cells were used as the control ( Figure 1E). After reviewing the current literature, we selected 30 novel upregulated proteins, such as FUNDC2 and CPNE1, and verified these genes by qRT-PCR. We found that 20 of them were consistent with the mass spectrometry results ( Figure 1F).

### KCTD14, CPNE1 and FUNDC2 are associated with cell proliferation and metastasis

**Figure 2:**
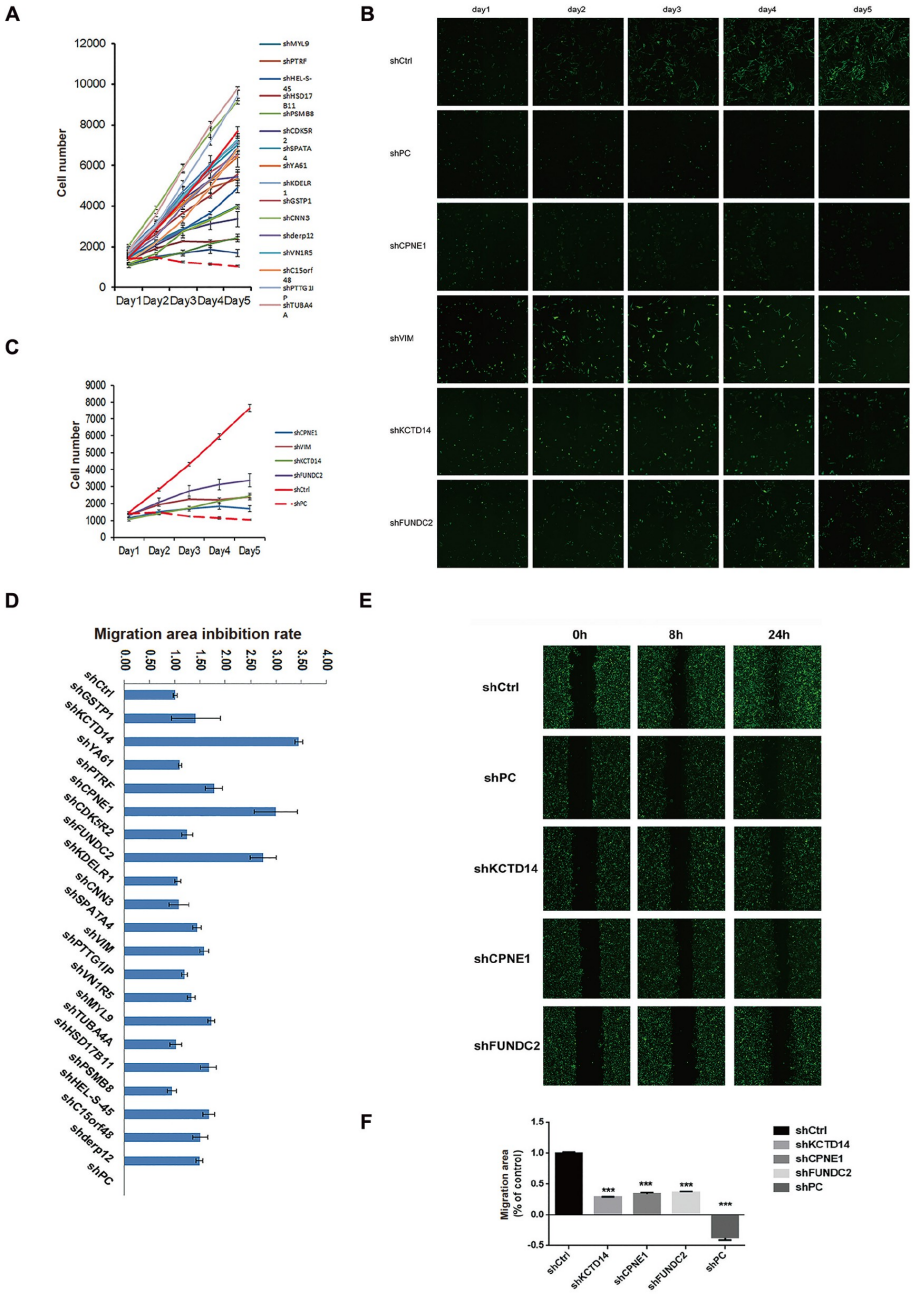
Figure 2 Identification of genes associated with cell proliferation and metastasis by high-content screening (A) Twenty genes with significant proliferation inhibition phenotypes were detected by high-content screening. (B) Representative proliferation images of shCtrl, shPC, shCPNE1, shVIM, shKCTD14 and shFUNDC2 (shCtrl was the negative control, shPC was the positive control). (C)The cell number statistical diagram of shCtrl, shPC, shCPNE1, shVIM, shKCTD14 and shFUNDC2. Data are expressed as the mean±SD. (D) The migration area inhibition rate of 20 genes was statistically analyzed by high-content screening. (E) Representative migration images of shCtrl, shPC, shCPNE1, shKCTD14 and shFUNDC2 at 0 h, 8 h and 24 h in the scratch assay. Graphs were taken and scanned by Celigo (Celigo 96 Wounding, Nexcelom). (F) The migration area statistical diagram of shCtrl, shPC, shCPNE1, shKCTD14 and shFUNDC2. Data are expressed as the mean±SD. *** P<0.001.

We examined the effect of these 20 differentially expressed genes on cell proliferation through high-content screening (HCS) ( Figure 2A). The results showed that the genes with obvious proliferation-inhibiting phenotypes were *CPNE1*, *VIM*, *KCTD14* and *FUNDC2* among the 20 genes ( Figure 2B,C). The scratch experiment of HCS was used to compare the cell migration ability of each group. We detected the ability of 20 genes to promote cell migration and calculated the migration inhibition rate ( Figure 2D). KCTD14, CPNE1 and FUNDC2 significantly promoted cell migration ( Figure 2E,F).

### The differential expressions and clinicopathological significance of FUNDC2 and KCTD14 in breast cancer

**Figure 3:**
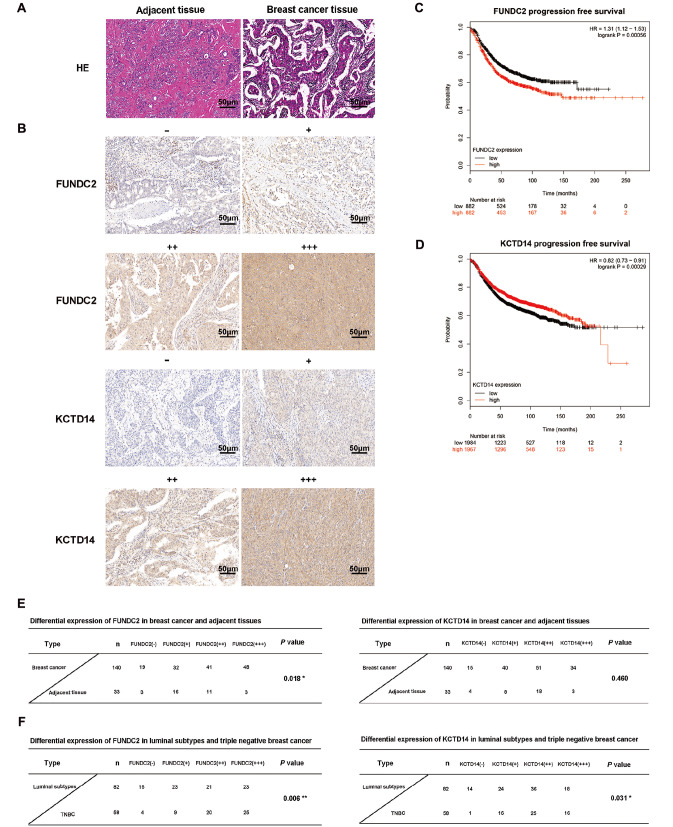
Figure 3 The differential expressions and clinical pathological significance of FUNDC2 and KCTD14 in breast cancer (A) Representative HE staining images of paracancerous tissue and breast cancer tissue. (B) Representative immunohistochemical images of different staining intensities of KCTD14 and FUNDC2. According to the staining range and intensity of the whole tissue specimen, the protein expression was divided into four grades: (–), (+), (++) and (+++). (C) Progression-free survival curve of FUNDC2 mRNA expression in TNBC patients. Red represents FUNDC2 high expression, black represents FUNDC2 low expression, data from Kaplan-Meier Plotter online analysis tool. (D) Progression-free survival curve of KCTD14 mRNA expression in TNBC patients. Red represents high KCTD14 expression, and black represents low KCTD14 expression. Data were obtained from the Kaplan-Meier Plotter online analysis tool. (E) Differential expression of FUNDC2 and KCTD14 in breast cancer and adjacent tissues. (F) Differential expression of FUNDC2 and KCTD14 in the luminal and triple-negative subtypes of breast cancer. Scale bar: 50 μm.

Vimentin (VIM) has been demonstrated to be a mesenchymal marker that is expressed in some cancer types, including breast cancer [28], and its effect on migration is not significant. CPNE1 has been reported in HER2-positive and luminal A breast cancer and TNBC [ 29, 30] . Therefore, we focused our research on KCTD14 and FUNDC2, which might be novel targets in breast cancer, especially TNBC. Immunohistochemical EliVision method was used to detect the expressions of KCTD14 and FUNDC2 in 82 luminal subtype breast cancer tissues, 58 TNBC paraffin tissue samples and 33 nontumor benign lesions ( Figure 3A,B). There was a significant difference in the expression of FUNDC2 between breast cancer and nontumor benign lesions ( *P*<0.05). However, KCTD14 expression did not differ ( Figure 3E). The expressions of FUNDC2 and KCTD14 in luminal subtype breast cancer tissues and TNBC tissues were statistically different ( *P*<0.05; Figure 3F). FUNDC2 was negatively correlated with ER, PR and HER2 in breast cancer ( *P*<0.05; Table 1). KCTD14 did not appear to be associated with ER, PR or HER2 ( *P*>0.05; Table 2). Meanwhile, to explore whether FUNDC2 or KCTD14 is related to the prognosis of TNBC patients, the Kaplan-Meier Plotter online analysis tool was used. It was found that there was a significant difference in progression-free survival between patients with high FUNDC2 expression and low FUNDC2 expression in TNBC ( *P*=0.00056). FUNDC2 expression was negatively correlated with TNBC progression-free survival ( Figure 3C). However, KCTD14 expression was positively correlated with TNBC progression-free survival ( *P*=0.00029; Figure 3D). Considering the consistency of the results, FUNDC2 might be a better target in TNBC than KCTD14, and thus, we focused on the role of FUNDC2 in TNBC.

**Table:** **Table 1** Correlation analysis of FUNDC2 expression with ER, PR, and HER2 in breast cancer

	*n*	FUNDC2 expression	*P* value	*r*
	–	+	++	+++		
ER						0.018*	–0.200
–	58(100%)	4(6.9%)	9(15.5%)	20(34.5%)	25(43.1%)		
+	26(100%)	3(11.5%)	9(34.6%)	5(19.2%)	9(34.6%)		
++	10(100%)	5(50.0%)	3(30.0%)	1(10.0%)	1(10.0%)		
+++	46(100%)	7(12.5%)	11(23.9%)	15(32.6%)	13(28.3%)		
PR						0.004*	–0.240
–	66(100%)	4(6.1%)	11(16.7%)	23(34.8%)	28(42.4%)		
+	25(100%)	4(16.0%)	8(32.0%)	5(20.0%)	8(32.0%)		
++	10(100%)	3(30.0%)	4(40.0%)	1(10.0%)	2(20.0%)		
+++	39(100%)	8(20.5%)	9(23.1%)	12(30.8%)	10(25.6%)		
HER2						0.000*	–0.332
–	81(100%)	7(8.6%)	12(14.8%)	27(33.3%)	35(43.2%)		
+	26(100%)	3(11.5%)	8(30.8%)	8(30.8%)	7(26.9%)		
++	26(100%)	6(23.1%)	9(34.6%)	6(23.1%)	5(19.2%)		
+++	7(100%)	3(42.9%)	3(42.9%)	0(0.0%)	1(14.3%)		

* *P*<0.05.

**Table:** **Table 2** Correlation analysis of KCTD14 expression with ER, PR, HER2 in breast cancer

	*n*	KCTD14 expression	*P* value	*r*
	–	+	++	+++		
ER						0.085	–0.146
–	58(100%)	1(1.7%)	16(27.6%)	25(43.1%)	16(27.6%)		
+	26(100%)	4(15.4%)	8(30.8%)	10(38.5%)	4(15.4%)		
++	10(100%)	1(10.0%)	5(50.0%)	2(20.0%)	2(20.0%)		
+++	46(100%)	9(19.6%)	11(23.9%)	14(30.4%)	12(26.1%)		
PR						0.070	–0.153
–	66(100%)	2(3.0%)	21(31.8%)	26(39.4%)	17(25.8%)		
+	25(100%)	4(16.0%)	4(16.0%)	10(40.0%)	7(28.0%)		
++	10(100%)	0(0.0%)	5(50.0%)	2(20.0%)	3(30.0%)		
+++	39(100%)	9(23.1%)	10(25.6%)	13(33.3%)	7(17.9%)		
HER2						0.053	–0.164
–	81(100%)	5(6.2%)	22(27.2%)	32(39.5%)	22(27.2%)		
+	26(100%)	1(3.8%)	10(38.5%)	9(34.6%)	6(23.1%)		
++	26(100%)	8(30.8%)	8(30.8%)	6(23.1%)	4(15.4%)		
+++	7(100%)	1(14.3%)	0(0.0%)	4(57.1%)	2(28.6%)		

### Knockdown of *FUNDC2* inhibits the proliferation, migration and invasion of TNBC cells

**Figure 4:**
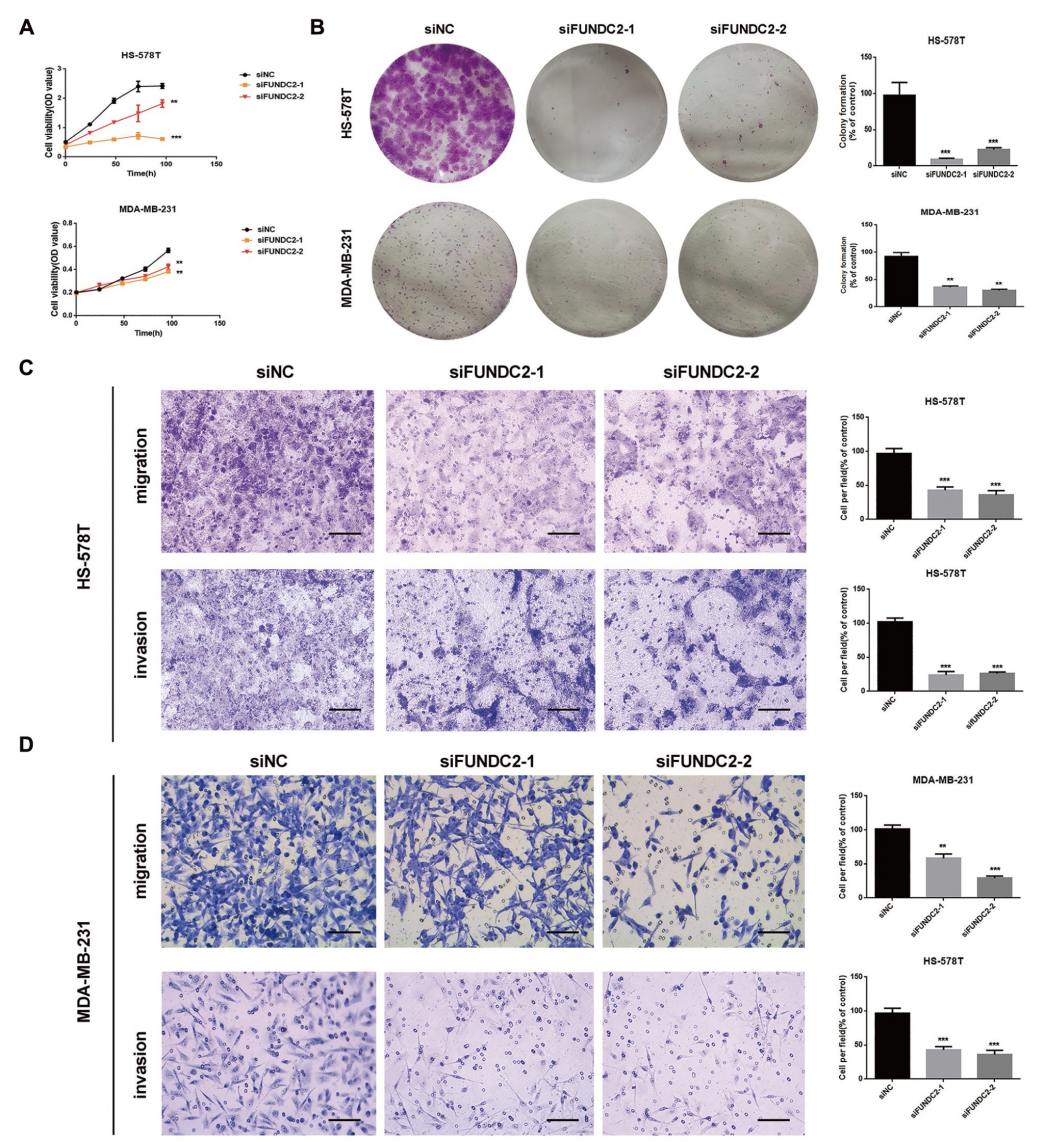
Figure 4 Knockdown of *FUNDC2* inhibits the proliferation, migration and invasion of TNBC cells (A) MDA-MB-231 and HS-578T cells were transfected with siNC (negative control) or siFUNDC2 (FUNDC2 siRNA), and cell viability at 0, 24, 48, 72 and 96 h was detected by the MTS assay. (B) Cells were treated as described in (A), 1000 viable cells were seeded in 6-well plates, and the number of cell clones was counted after 14 days. Left, representative photos of three independent experiments; right, statistical charts. Data are presented as the mean±SD. Student’s t test. ** P<0.01, *** P<0.001. (C,D) The invasion and migration abilities of MDA-MB-231 and HS-578T cells were detected by transwell assay. Cells were treated as described above , and then 2×10 5 or 1×10 5 viable cells were added cultured for 48 h, then fixed and stained for imaging. Left, representative images. Scale bar: 100 μm ( n=3, independent experiments). Right, statistical charts. Data are presented as the mean±SD. Student’s t test. ** P<0.01, *** P<0.001.

To investigate the role of FUNDC2 in TNBC, the effects of *FUNDC2* silencing on the proliferation of MDA-MB-231 and HS-578T cells were detected by MTS assay and plate clone formation assay. We found that compared with the negative control group (NC group), the proliferation ability of the siFUNDC2 group was significantly inhibited ( *P*<0.001), suggesting that FUNDC2 can promote the proliferation of TNBC cells ( Figure 4A,B). Transwell assays were used to detect the effects of FUNDC2 on the migration and invasion of MDA-MB-231 and HS-578T cells. The results showed that compared with the negative control cells, the migration and invasion abilities of TNBC cells were significantly suppressed after *FUNDC2* knockdown ( Figure 4C,D).

### Knockdown of *FUNDC2* inhibits TNBC by inactivating the AKT/GSK3β/GLI1 pathway

**Figure 5:**
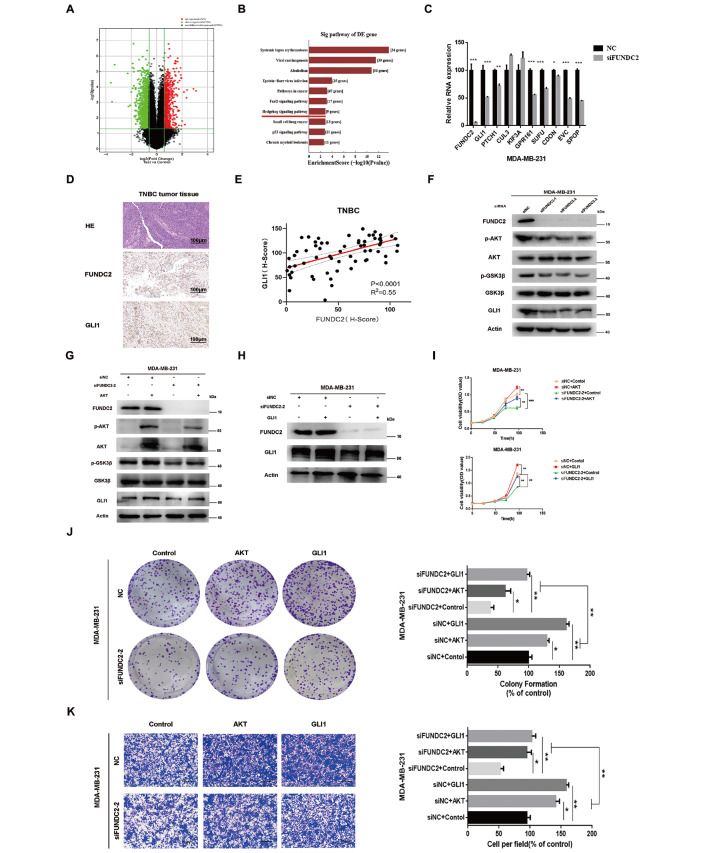
Figure 5 Knockdown of *FUNDC2* inhibits TNBC by inactivating the AKT/GSK3β/GLI1 pathway (A) Volcanic clustering analysis of gene differences after FUNDC2 silencing. The red dots represent genes with high expression, and the green dots represent genes with low expression. P<0.05, log 2(fold change) siFUNDC2 vs siNC≥1. (B) Histogram of KEGG enrichment analysis of differentially expressed genes after FUNDC2 silencing. (C) Seven genes in the Hedgehog pathway were significantly downregulated after silencing of FUNDC2 in MDA-MB-231 cells, Data are presented as the mean±SD, n=3 independent experiments. Statistical significance was determined by Student’s t test. * P<0.05, ** P<0.01. *** P<0.001. (D) Representative images of HE staining and GLI1 and FUNDC2 IHC staining in TNBC tumor tissue. (E) Correlation between FUNDC2 and GLI1 expressions in the TNBC tissue microarray. X-axis: FUNDC2 (H-score), Y-axis: GLI1 (H-Score). The H-score was calculated by multiplying the proportion and intensity. Pearson correlation test ( n=66, R 2=0.55, P<0.0001). (F) MDA-MB-231 cells were transfected with siNC or siFUNDC2 for 48 h, and then cell lysates were collected for immunoblotting. The levels of p-AKT, AKT, p-GSK3β, GLI1, FUNDC2 and GSK3β were analyzed by western blot analysis. (G,H) MDA-MB-231 cells were transfected with siNC+AKT or siNC +GLI1 and siFUNDC2+AKT or siFUNDC2 +GLI1 for 48 h, and then cell lysates were collected for immunoblotting. ( I) MDA-MB-231 cells were transfected with siNC+AKT or siNC+GLI1 and siFUNDC2 +AKT or siFUNDC2+GLI1, and cell viability at 0 h, 24 h, 48 h, 72 h, 96 h was detected by the MTS assay. Student’s t test. ** P<0.01, *** P<0.001. (J) Cells were treated as described in H, and then 1000 viable cells were seeded in 6-well plates. The number of cell clones was counted after 14 days. Left, representative photos of three independent experiments; right, statistical charts. Data are presented as the mean±SD. Student’s t test. * P<0.05, ** P<0.01. (K) The migration abilities of MDA-MB-231 cells were detected by transwell assay. Cells were treated as described for (H), and then 1×10 5 viable cells were cultured for 48 h, fixed and stained for imaging. Left, representative images. Scale bar: 100 μm ( n=3, independent experiments). Right, statistical charts. Data are presented as the mean±SD. Student’s t test. * P<0.05, ** P<0.01.

To reveal the underlying mechanism of FUNDC2 in TNBC, we knocked down *FUNDC2* in MDA-MB-231 cells and screened differentially expressed genes by genome-wide microarray. We found that 729 genes were significantly upregulated and 1298 genes were significantly downregulated ( Figure 5A). Moreover, differentially downregulated genes were mainly enriched in 10 pathways, including the Hedgehog signaling pathway, cancer pathway, FoxO signaling pathway and p53 signaling pathway, according to KEGG enrichment analysis ( *P*<0.05; Figure 5B).

The Hedgehog pathway is a key driver of TNBC development and can promote TNBC proliferation, invasion and migration, angiogenesis and stem cell reprogramming [ 31, 32] . Therefore, we focused on demonstrating whether the effect of FUNDC2 knockdown is mediated by the Hedgehog pathway in MDA-MB-231 cells. We found that compared with the control group, 7 genes of the Hedgehog pathway were significantly downregulated after *FUDNC2* silencing, of which GLI1, the key transcriptional activator of the Hedgehog pathway, was remarkably downregulated ( *P*<0.05; Figure 5C). We collected 78 groups of TNBC and used IHC staining to determine the expressions of FUNDC2 and GLI1 in TNBC tissues. There was a significant positive correlation between FUNDC2 and GLI1 in TNBC tumor tissues ( *R*=0.552, *P*<0.001; Figure 5D,E).

To explore how FUNDC2 regulates GLI1, western blot analysis was performed on MDA-MB-231 cells, and the results showed that the expressions of p-AKT/p-GSK3β and GLI1 were significantly inhibited after FUNDC2 downregulation, while the total protein levels of AKT and GSK3β were not obviously changed ( Figure 5F and Supplementary Figure S1A). Furthermore, we conducted rescue experiments to confirm the role of FUNDC2 in promoting TNBC progression through the AKT/GSK3β signaling pathway. Overexpression of AKT or GLI1 rescued the effect of FUNDC2 siRNA transfection on modifying p-AKT/p-GSK3β and GLI1 protein levels ( Figure 5G,H). Further verification was achieved by gray-level analysis ( Supplementary Figure S1B,C). The results of the MTS assay and plate colony formation assay showed that the proliferation abilities of MDA-MB-231 cells in the siNC+AKT and siNC+GLI1 groups were significantly higher than those in the siFUNDC2+AKT and siFUNDC2 +GLI1 groups ( Figure 5I,J). Transwell assays also showed that the migration abilities of MDA-MB-231 cells in the siFUNDC2 +AKT and siFUNDC2 +GLI1 groups were significantly inhibited compared with those in the siNC +AKT and siNC +GLI1 groups ( Figure 5K).

The above results showed that FUNDC2 might regulate the protein expression of GLI1, the Hedgehog signaling pathway activator, through the AKT/GSK3β signaling pathway, thus regulating the proliferation, invasion and migration of TNBC cells.

### *FUNDC2* silencing suppresses the growth of xenografted MDA-MB-231 cells in BALB/C-nude mice

**Figure 6:**
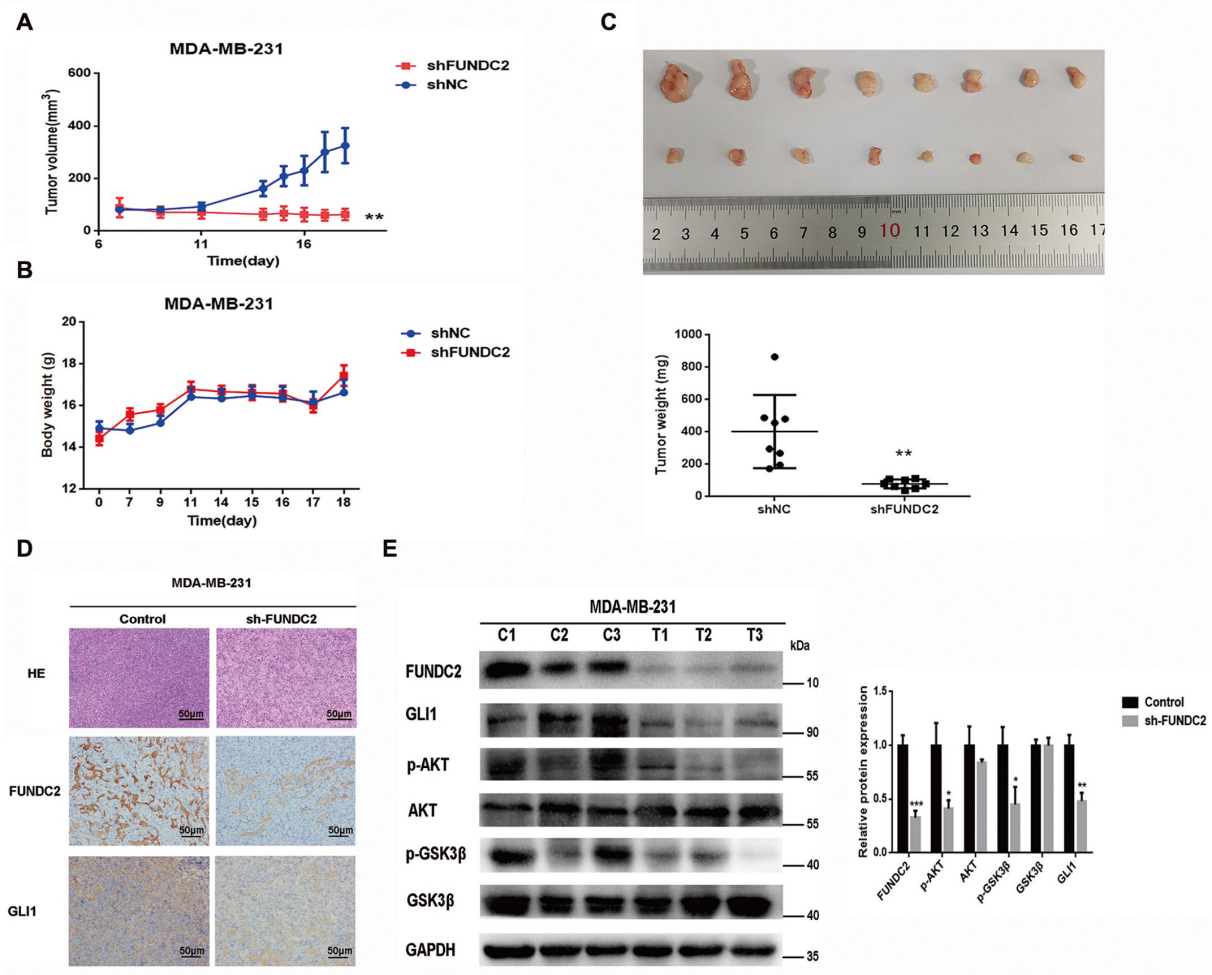
Figure 6 *FUNDC2* silencing suppresses the growth of xenografted MDA-MB-231 cells in BALB/C-nude mice (A) Subcutaneous tumor growth curve of MDA-MB-231 cells in BALB/C-nude mice. The shFUNDC2 stable cell line was established by infecting MDA-MB-231 cells with shFUNDC2 lentivirus. Stable cell lines (2×10 6 cells) and matrigel were mixed and xenografted subcutaneously into BALB/C-nude mice. The tumor volume (mm 3) was measured one week later. (B) Body weight curves of BALB/C-nude mice during the experiment. (C) Representative images of subcutaneous tumor size in mice at the end of the experiment (upper) and the statistical chart of tumor weight (lower). (D) Representative images of HE staining and immunohistochemical staining of FUNDC2 and GLI1 expressions. Scale bar: 50 μm. (E) Immunoblotting was performed on xenografted tumor tissues after the experiment. The tumor tissues were selected randomly. * P<0.05, ** P<0.01, *** P<0.001.

To further study the role of FUNDC2 *in vivo*, MDA-MB-231 cell lines stably expressing shFUNDC2 were transplanted subcutaneously into nude mice, and a negative control group was also established. Tumor volume and body weight of mice were measured on the 7th day of growth and continued until the 18th day. Based on the tumor growth curve and body weight change curve, the tumor volume of the shFUNDC2 group was significantly lower than that of the NC group ( Figure 6A), but there was no significant difference in body weight ( Figure 6B). After tumor dissociation, the tumor weight of the NC group was significantly higher than that of the shFUNDC2 group ( Figure 6C). Immunohistochemical analysis of FUNDC2 and GLI1 expressions in tumor tissues showed a positive correlation between them ( Figure 6D). Immunoblotting of xenograft tissues from mice demonstrated that *FUNDC2* silencing suppressed the growth of MDA-MB-231 tumors by inactivating the AKT/GSK3β/GLI1 pathway, which was consistent with the *in vitro* data ( Figure 6E).

## Discussion

Triple-negative breast cancer accounts for approximately 10%–20% of all breast cancers [ 2, 5, 6] , but it has a very high degree of malignancy with strong invasive and metastatic ability, poor prognosis and easy recurrence [ 33, 34] . Clinically, the commonly used treatments for TNBC are chemotherapy and surgery [ 7, 35] . Due to its special molecular type, there are currently no special targeted drugs for this type of breast cancer. Long-term clinical studies have shown that the efficacy of various chemotherapy regimens is not good. Therefore, to improve the therapeutic efficacy and prognosis of TNBC, it is necessary to explore novel targeted therapies.

We screened 20 novel upregulated proteins using ITRAQ technology combined with qRT-PCR validation in luminal A cells and TNBC cells ( Figure 1). Notably, KCTD14 was also a candidate target in the preliminary screening. However, we found that the expression of KCTD14 was not significantly different between breast cancer tissue and adjacent tissue, and KCTD14 expression was positively correlated with TNBC progression-free survival ( Figure 3D,E). This is not consistent with the results of HCS screening in TNBC. The problem may be due to an insufficient size of the research samples. A limited amount of literature exists regarding the role of KCTD14 in normal physiological function or tumorigenesis. Further research is needed to determine the exact role of KCTD14 and the underlying mechanism by which it functions.

We found that FUNDC2 may be a potential therapeutic target of TNBC by HCS screening and clinical sample analysis ( Figures 2 and 3). A few reports on FUNDC2 have been published. Two studies in platelets showed that via the AKT signaling pathway FUNDC2 regulates platelet activation and supports platelet survival, and that FUNDC2 is a novel mitochondrial outer membrane protein that binds to PIP3 directly [ 11, 12] . FUNDC1 is a highly conserved paralogue of FUNDC2, which regulates mitophagy and inflammation [36]. It has been shown that the LIR motifs present in FUNDC1 are essential for FUNDC1-mediated mitophagy in platelets [ 37, 38] . However, *FUNDC2*-KO platelets or HeLa cells did not exhibit mitophagy, as FUNDC2 does not contain the LIR motif [12]. FUNDC2 has been reported in human hepatocellular carcinoma [13], but it is worth noting that the role of FUNDC2 in breast cancer and other tumor types has not been studied. Currently, there are no effective targeted drugs for FUNDC2. Our results indicated that FUNDC2 expression in patients with triple-negative breast cancer was significantly higher than that in patients with luminal subtype breast cancer, and FUNDC2 expression was positively correlated with TNBC progression-free survival ( Figure 3C,F). It was also negatively correlated with ER, PR and HER2 ( *P*<0.05; Table 2). Thus, we focused on FUNDC2.

It is evident that FUNDC2 is involved in promoting TNBC proliferation, invasion and migration ( Figure 4). FUNDC2 inhibited tumor growth in a subcutaneous tumorigenesis experiment in mice ( Figure 6). Then, how does FUNDC2 promote the proliferation, invasion and metastasis of TNBC? The structure of FUNDC2 differs from that of FUNDC1, and FUNDC2 is a novel mitochondrial outer membrane protein. The function of FUNDC2 in cancer, however, is poorly understood. In this study, we found that the Hedgehog signaling pathway was closely related to FUNDC2 by genome-wide microarray screening and KEGG enrichment analysis ( Figure 5A,B). Therefore, we further verified this finding by immunoblotting and TMA, and the results showed that there was a positive correlation between FUNDC2 and GLI1, the activator of the Hedgehog signaling pathway ( Figure 5F). Previous reports showed that FUNDC2/PIP3 enhanced the phosphorylation of AKT on mitochondria, and AKT phosphorylation promoted GSK3β phosphorylation, while activation of GSK3β inhibited the GLI1/Hedgehog pathway [ 11, 22] . Next, by immunoblotting, we demonstrated that *FUNDC2* silencing in the triple-negative breast cancer cell line MDA-MB-231 can indeed reduce the expression of AKT and GSK3β phosphorylation (p-AKT and p-GSK3β) at the protein level, which was further validated in xenografted MDA-MB-231 cells in BALB/C-nude mouse tissue ( Figures 5D and 6E). This inhibitory effect could be reversed by AKT or GLI1 overexpression ( Figure 5G‒K). Despite this, when FUNDC2 was silenced, there were 9 other pathways except the Hedgehog signaling pathway, which confirmed that other functions of FUNDC2 have not yet been revealed. The exact mechanism by which FUNDC2 regulates other targets of the Hedgehog signaling pathway, such as PTCH1 and CUL3, is still unknown. Whether FUNDC2 regulates TNBC through mitochondrial fusion and other pathways remains to be further explored.

In summary, the regulatory mechanism of triple-negative breast cancer is complex. We preliminarily confirmed that the mitochondrial outer membrane protein FUNDC2 may promote the development of triple-negative breast cancer through the AKT/GSK3β/GLI1 signaling axis. Therefore, FUNDC2 seems to be a potential target for TNBC.

## Supporting information

23169Figure_S1

23169Supplementary_Table_S1

23169Supplementary_Table_S2

## Supplementary Data

Supplementary data is available at *Acta Biochimica et Biophysica Sinica* online.
