# Supplementary figures and images for: FUNDC2, a mitochondrial outer membrane protein, mediates triple-negative breast cancer progression via the AKT/GSK3β/GLI1 pathway: FUNDC2 mediates TNBC progression via the AKT/GSK3β/GLI1 pathway
Source: Acta Biochim Biophys Sin (Shanghai). 2023 Sep 11;55(11):1770–83. doi: 10.3724/abbs.2023142 (PMC10679879; doi:10.3724/abbs.2023142)

Figure S1

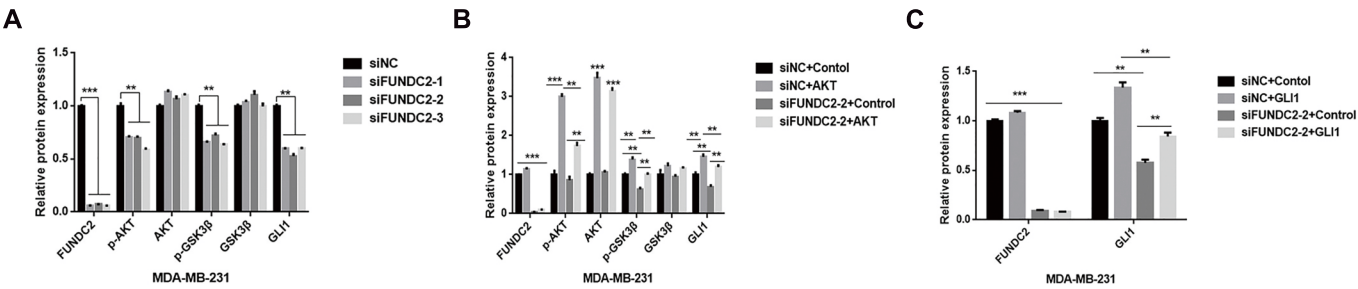

**A,B,C**, A gray value statistical graph of various protein changes is shown in the graph , \*P < 0.05, \*\*P < 0.01, \*\*\*P < 0.001.

Supplement: 23169Figure_S1 [file 23169Figure_S1.pdf]
