# Supplementary material for: FUNDC2, a mitochondrial outer membrane protein, mediates triple-negative breast cancer progression via the AKT/GSK3β/GLI1 pathway: FUNDC2 mediates TNBC progression via the AKT/GSK3β/GLI1 pathway
Source: Acta Biochim Biophys Sin (Shanghai). 2023 Sep 11;55(11):1770–83. doi: 10.3724/abbs.2023142 (PMC10679879; doi:10.3724/abbs.2023142)
Supplement: 23169Supplementary_Table_S1 [file 23169Supplementary_Table_S1.pdf]

Supplementary Table s1. The sequences of shRNAs used in this study

Targets	Sequences (5'→3')
CPNE1-shRNA1	TAGAGGCCAGAAACCTAGATA
CPNE1-shRNA2	CACACAACTGGTCTCATACTT
CPNE1-shRNA3	GAGTACTCCTTTCTGGACTAT
FUNDC2-shRNA1	GATCCGTAAGAGCAATCAGAT
FUNDC2-shRNA2	CACAGGTTTCATATTCCAGAA
FUNDC2-shRNA3	GAATCTGGACCTTCAGCAGAA
KCTD14-shRNA1	CAAGGTATTCTCCAAGTTCTA
KCTD14-shRNA2	AGAAGCCATAACAGCACGGAA
KCTD14-shRNA3	GTTCTACGAAATCAAGCCTTT
CNN3-shRNA1	GATTACCAATATAGCGACCAA
CNN3-shRNA2	GACCACAATTAGTCTGCAGAT
CNN3-shRNA3	GCAGAAGAAGATCTTCGCAAT
PTRF-shRNA1	GAGCATCAGCAAATCGCTGAA
PTRF-shRNA2	GTGGAGGTTGAGGAGGTTATT
PTRF-shRNA2	CCGCAACTTTAAAGTCATGAT
CDK5R2-shRNA1	CCGGAACCAAGCACTGGACTA
CDK5R2-shRNA2	GCAGGTCTTTCAAGACCTCAA
CDK5R2-shRNA3	CCGACGCTGCTATCGCCTCAA
KDELR1-shRNA1	GATTTCTTCTACCTCTATA
KDELR1-shRNA2	TCTATCTCTTCAACTGGAT
KDELR1-shRNA3	TACTTACGATGGGAACCAT
SPATA4-shRNA1	AGCTGGACAACATTCTTAT
SPATA4-shRNA2	ATGAACTTAAAGCGGAGTT
SPATA4-shRNA3	CCATCTCTAGCCATAATAA
VIM-shRNA1	CAGGTTATCAACGAAACTT
VIM-shRNA2	CTGGTTGATACCCACTCAA
VIM-shRNA3	AGGATGAGATTCAGAATAT
VN1R5-shRNA1	TCACATACTGGGTGGACTT
VN1R5-shRNA2	AGGCTACATGGTGATTATT
VN1R5-shRNA3	CAGGATGATCTTAGGTATA
TUBA4A-shRNA1	GGACAACGAAGCAATCTAT
TUBA4A-shRNA2	TCTCTGTTGACTATGGCAA
TUBA4A-shRNA3	CCGTGGTCACTATACCATT
PSMB8-shRNA1	ACGTTAAGTCCAAGGAGAA
PSMB8-shRNA2	TGGGTGAAAGTAGAAAGTA
PSMB8-shRNA3	TCTCTATGGGCAGTATGAT
C15orf48-shRNA1	AACCCATTGAAGAGTTGCA
C15orf48-shRNA2	TGTGTATTCTCTTTGGAAA

C15orf48-shRNA3	AGGAACTCATTCCCTTGGT
PTTG1IP-shRNA1	CTTGCAGACTGGTGACCTT
PTTG1IP-shRNA2	AGGGCTCGGTTGTGAAATA
PTTG1IP--shRNA3	CCCGTATGCTAGATTTGAA
MYL9-shRNA1	TTGATAAGAAAGGCAACTT
MYL9-shRNA2	CACATCCAATGTCTTCGCAAT
MYL9-shRNA3	ACCGTGATGGCTTCATTGACA
HSD17B11--shRNA1	GTTAAGTTTGATGCAGTTA
HSD17B11--shRNA2	GTAGTCTATACATCAGATT
HSD17B11--shRNA3	TGGTTCTCTGGGATATAAA
HEL-S-45--shRNA1	TGAAGATGGGATCCTAGACAT
HEL-S-45--shRNA2	ATGAACATGGGCAGTGACTTT
HEL-S-45--shRNA3	GGGCAGTGACTTTGACGTCTT
derp12-shRNA1	CAGCTAGAGTATTTGAAGAAG
derp12-shRNA2	CTGCTTTAGGGTCTGAAGTTT
derp12-shRNA3	TTGGTGATGTAACTGGTTCAA
