# Supplementary material for: FUNDC2, a mitochondrial outer membrane protein, mediates triple-negative breast cancer progression via the AKT/GSK3β/GLI1 pathway: FUNDC2 mediates TNBC progression via the AKT/GSK3β/GLI1 pathway
Source: Acta Biochim Biophys Sin (Shanghai). 2023 Sep 11;55(11):1770–83. doi: 10.3724/abbs.2023142 (PMC10679879; doi:10.3724/abbs.2023142)
Supplement: 23169Supplementary_Table_S2 [file 23169Supplementary_Table_S2.pdf]

Supplementary Table S2. The sequences of primers used this study

Gene	Forward primer (5'→3')	Reverse primer (5'→3')
PLEC	TGCCTACTTTCAGTTCTTC	CGATCACAACTGTATTTCCT
GSTP1	GTCCAATACCATCCTGCGTCAC	CATCCTTGCCCGCCTCATAG
KRT85	ACACCTCGGTCATAGTCAA	CTGGCAACATCGTCATACT
VIM	AATGGCTCGTCACCTTCG	CTAGTTTCAACCGTCTTAATCAG
TGM2	CAAGGCCCGTTTTCCACTAAG	GAGGCGATACAGGCCGATG
DDX59	TGATGGCAAAAGTTGTGTGGC	AGGGCATGATTCGCTGATGTG
SPATA4	TGGTTTCCAGGTCTACAGT	GTAAAACAGGGACAACTCT
EEF1A1	CCCAGGACACAGAGACTTTATC	CAACACCAGCAGCAACAATC
CDK5R2	CAAACCTGGTGTTCGTGTA	GTTGCCCATGTAGGAGTAG
KCTD14	TCTTTGGTGAGCAGGTGTC	ATGTCCATCTCCAGGCAGT
PTRF	GGGCCGTAGACCAGATCCA	CTTGCTCACCGTATTGCTCGT
PTTG1IP	CTTCCCTTTGTAAATTGAGC	AGCATACGGGTTTTCTTCT
C15orf48	CTGATGAAAAGGAAGGAAC	TTTGAGGTACAGTAGGGTC
HSD17B11	TGTCTCGGTCCCCTTCTTA	ACCACTTCCTCAGGTTCCA
RMND5A	GGCCTCAAGCACGAGATCC	CTTTTCCAACCCGAGAAACACT
VN1R5	TAAACAAGGTGATGAGGGG	TTTGCCAAGGAGAAGATGC
NLRP8	CACAGGAAATGGGTGTTAG	GAATACTCATGCCAAGGAA
PSMB8	CTGGGTCCTACATTAGTGCCT	CCATTTCGCAGATAGTACAGC
TUBA4A	CACCACCTTCTTCTGTGAA	CACTGGGTCAATGATCTCC
KDELR1	ACGGTCTGGTTGATTTATAG	CAAGTAGTGGCTGGTGATG
FAM8A1	GGGTTTTAGTGATTCCTTC	AGCTGTTCGATTATGCTGA

FUNDC2	TGGTGGCGTAAGCTGTTCG	CCAGTCAACTTTGATGTACCCAG
CPNE1	ACCCACTCTGCGTCCTT	TGGCGTCTTGTTGTCTATG
CNN3	TTACGGGACTAGGAGGCATCT	CCGGCTGTAATGTTAGCTTCTG
MYL9	AGGATGTGATTCGCAACGC	TTGAGGATGCGGGTGAACT
STON2	GAGAATGTGATGATCCGTT	TGGCAGTTCCCAGAGTTAC
POLR3A	TTTGACGCTGCCTACTTCG	GGTCCCTGTCAGCCTTGTG
DKFZp686D1968	GAGACGTGCCCATCGACCA	GCTGTCTTCCCGCGTGATC
YA61	GAGCTAGAGCTGCTGCTTA	CTTGATCTTCGTTGTATTGG
derp12	GACAAGATTTAGGTGGGACT	ACTTCAACACCTGCATTAG
GLI1	GAAGCCGAGCCGAGTATC	GGTGAGTAGACAGAGGTTGG
PTCH1	CCACAGAAGCGCTCCTACA	CTGTAATTTCGCCCCTTCC
CDON	TGGAAATGAAGCCCCTCAGT	GACGCTCTCCTCCGGCA
CUL3	TCGACAGCTCACACTCCAGCAT	GTGCTTCCGTGTATTAGAGCCAG
EVC	ACTTCAGCACCGTGGACACTTTC	TCTCCTGGACTTCCTGCCTCAAG
KIF3A	CTGATATCAGTGGGTCAGAGGA	TCCAGCAAAGACTGATGCTCT
SUFU	CTCCTGACTTGTAACAGGGACC	AGTTGCAAGCAGGGAGAAAA
SPOP	GCCCCGTAGCTGAGAGTTG	ACTCGCAAACACCATTTCAGT
GPR161	TGGATCTTTGGTGTAGTGTGGT	ATGACCCCGAGGGTTAGCAT
GAPDH	AGCCTCAAGATCATCAGCAATGCC	TGTGGTCATGAGTCCTTCCACGAT
